# Decreased Density of Perineuronal Net in Prelimbic Cortex Is Linked to Depressive-Like Behavior in Young-Aged Rats

**DOI:** 10.3389/fnmol.2020.00004

**Published:** 2020-01-28

**Authors:** Zhoulong Yu, Na Chen, Die Hu, Wenxi Chen, Yi Yuan, Shiqiu Meng, Wen Zhang, Lin Lu, Ying Han, Jie Shi

**Affiliations:** ^1^National Institute on Drug Dependence and Beijing Key Laboratory of Drug Dependence, Peking University, Beijing, China; ^2^Department of Pharmacology, School of Basic Medical Sciences, Peking University Health Science Center, Beijing, China; ^3^Peking University Sixth Hospital, Peking University Institute of Mental Health, NHC Key Laboratory of Mental Health (Peking University), National Clinical Research Center for Mental Disorders (Peking University Sixth Hospital), Beijing, China; ^4^Peking-Tsinghua Center for Life Sciences and PKU-IDG/McGovern Institute for Brain Research, Peking University, Beijing, China; ^5^The State Key Laboratory of Natural and Biomimetic Drugs, Peking University, Beijing, China; ^6^The Key Laboratory for Neuroscience of the Ministry of Education and Health, Peking University, Beijing, China

**Keywords:** perineuronal nets, depression, CUMS, prelimbic cortex, neurocan, aggrecan

## Abstract

Perineuronal nets (PNNs) are condensed extracellular matrix (ECM) structures regulating developmental plasticity and protecting neurons against oxidative stress. PNN abnormalities have been observed in various psychiatric disorders such as schizophrenia and bipolar disorder, but the relationship between PNN density and depression still remains unclear. In the present study, we examined the density and components of PNNs including aggrecan, neurocan and Tenascin-R in the prelimbic cortex (PrL) after chronic unpredictable mild stress (CUMS). We found that depressive-like behaviors were induced after 30 days of CUMS accompanied by decreases in PNN^+^ cell density and aggrecan expression in the PrL. In addition, rats subjected to 20 days of CUMS were separated into vulnerable and resilient subpopulations that differ along several behavioral domains. Consistently, the density of PNNs and the expression level of neurocan in the vulnerable group were decreased compared to control and resilient groups. Finally, we examined individual differences based on locomotion in a novel context and classified rats as high responding (HR) and low responding (LR) phenotypes. The density of PNNs and the expression level of neurocan in the LR group were lower than the HR group. Moreover, the LR rats were more susceptible to depressive-like behaviors compared with HR rats. Altogether, these results suggest that the density of PNNs in the PrL is associated with depressive-like behaviors in young-aged rats, and it may serve as a potential endophenotype or therapeutic target for depression.

## Introduction

Depression is a debilitating psychiatric disorder that causes disability and suicide worldwide (Moussavi et al., 2007; Phillips et al., 2009). Stressful life events are recognized as an important risk factor for psychiatric disorders, particularly depression and anxiety (Kendler et al., 1999, 2004). An individual’s response to stress is determined by both genetic and environmental elements and their complex interactions. Rodent models such as chronic unpredictable mild stress (CUMS) and social defeat are commonly utilized to characterize the biology of individual variations in susceptibility to stress (Krishnan et al., 2007; Bergström et al., 2008; Taliaz et al., 2011). A vast literature also describes that the individual differences in response to stress or a novel environment contribute to explain differential susceptibility to develop abnormal behaviors and are important predictors for the occurrence of neuropsychiatric disorders including depression (Armario and Nadal, 2013; Carreira et al., 2017; Weger and Sandi, 2018), but the biological basis underlying the resilience to depression is still poorly understood.

Perineuronal nets (PNNs) are specialized, condensed extracellular matrix (ECM) structures, composed largely of hyaluronan, link proteins, tenascin-R (Tn-R) and chondroitin sulfate proteoglycans (CSPGs), and arranged in lattice-like accumulations that surround the neurons (Celio et al., 1998; Deepa et al., 2006; Sorg et al., 2016). The main CSPGs in PNN are aggrecan, neurocan, brevican, versican and phosphacan (Deepa et al., 2006; Reichelt et al., 2019). PNN assembly is impaired in Tn-R knockout mice (Brückner et al., 2000). During development phases, the appearance of well-structured PNNs is considered to be a marker of maturation of parvalbumin (PV)-positive GABAergic interneurons in many cortical regions (McRae et al., 2007; Balmer et al., 2009; Baker et al., 2017). In adulthood, abnormalities in PNN structure and components can cause changes in synaptic plasticity and neuronal function (Bukalo et al., 2007; Miyata et al., 2012; Khoo et al., 2019). PNNs serve as a protective barrier and protect fast-spiking interneurons against oxidative stress and neurotoxins (Morawski et al., 2004, 2010, 2012; Cabungcal et al., 2013).

Previous studies have implicated the alterations in the density or composition of PNNs in a range of neuropsychiatric disorders such as epilepsy (Tewari et al., 2018), schizophrenia (Bitanihirwe and Woo, 2014; Enwright et al., 2016), bipolar disorder (Steullet et al., 2018), and drug addiction (Xue et al., 2014; Blacktop and Sorg, 2019), and these behavioral abnormalities might be improved after reversing the changes in the number of components of PNNs. Chronic stress during early life or adulthood alters the density of PNNs in the medial prefrontal cortex, and affects the structure and plasticity of inhibitory neurons, especially PV-expressing interneurons (Castillo-Gómez et al., 2017; de Araújo Costa Folha et al., 2017; Pesarico et al., 2019). Early life maltreatment or maternal immune activation caused developmental abnormalities in PNNs in the prefrontal cortex and basolateral amygdala (Paylor et al., 2016; Page and Coutellier, 2018; Santiago et al., 2018), which may be associated with the increased vulnerability to neuropsychiatric disorders such as anxiety. Chronic antidepressant treatments such as fluoxetine and venlafaxine have also been shown to alter PV- and PNN- positive cell density in cortical and hippocampal regions in adults (Ohira et al., 2013; Alaiyed et al., 2019) and these effects may be relevant to their antidepressant efficacy. Moreover, the sustained antidepressant effect of ketamine in the forced swim test (FST) is blocked by degrading PNNs in the ventral hippocampus (Donegan and Lodge, 2017). These findings suggest that PNNs may play a critical role in stress responses and the development of depression.

In the present study, we investigated the relationship between PNN integrity and individual differences in locomotor response to novelty and susceptibility to stress. Our findings revealed that the lower PNN numbers in the prelimbic cortex (PrL) may be a predictor for the development of depressive-like behaviors in young-aged rats.

## Materials and Methods

### Animals

Male Sprague–Dawley rats (8-week-old, 250 g–300 g) were obtained from the Laboratory Animal Center of Peking University Health Science Center. Animals were kept in standard cages in temperature (22 ± 2°C)—and humidity (50% ± 5%)—controlled rooms with a reverse 12 h/12 h light/dark cycle (lights on at 8:00 PM), and had free access to food and water. All experiments were approved by the National Institutes of Health Guide for the Care and Use of Laboratory Animals and were approved by the Biomedical Ethics Committee for Animal Use and Protection of Peking University.

### Chronic Unpredictable Mild Stress

The CUMS protocol was adapted from our previous studies (Li et al., 2018; Han et al., 2019). The rats were exposed to a variable sequence of mild and unpredictable stressors. A total of 10 different stressors were used, with two stressors per day. The stressors included cold for 1 h at 4°C, water deprivation for 24 h, vibration for 1 h, tilted cages (45°) for 24 h, forced cold swim for 5 min, crowding for 24 h, soiled bedding for 24 h, light/dark cycle reversal for 36 h, food deprivation for 24 h and tail clamp for 1 min.

### Sucrose Preference Test

The sucrose preference test (SPT) was performed to evaluate depressive-like behavior in rats. Rats were single-housed after stress and habituated to one bottle of water and one bottle of 1% sucrose for 2 days. The position of the two bottles was changed every 24 h during adaptation. After adaptation, the rats were deprived of water for 4 h and then subjected to the SPT. In the SPT, the rats were housed in individual cages and had free access to two bottles that contained 1% sucrose or water for 1 h. Sucrose preference was defined as sucrose consumption/(water consumption + sucrose consumption) × 100% during the test phase.

### Forced Swim Test

The FST was performed to evaluate depressive-like behavior in rats. Briefly, rats were placed individually in a water-filled cylinder (25 cm diameter × 65 cm height, 23–25°C) for 15 min of forced swimming in the adaptation session. After 24 h, the rats were placed in the cylinder again for a test session in 5 min. The time of immobility, defined as stopping struggling and remaining floating, was recorded. Immobility time (in seconds) was video recorded and analyzed using EthoVision XT 10 software.

### Novelty-Suppressed Feeding Test

The novelty-suppressed feeding test (NSFT) was performed to evaluate anxiety-like behavior in rats. Briefly, animals were food-restricted for 24 h, and then placed in the corner of an open field arena (75 cm × 75 cm × 40 cm), and a small amount of food was placed on a white paper square (10 cm × 10 cm) in the center of the cage. Each test lasted 10 min, and the latency to approach the food and begin eating was recorded as the latency time. Food consumption in the home cage in 5 min immediately after the test was assessed as a relative measure of hunger.

### Open Field Test

The open-field test (OFT) was used to assess baseline locomotor activity and categorize the rats into two different phenotypes: high-responding (HR) phenotype and low-responding (LR) phenotype. Male rats ware placed in a locomotor box (40 × 40 × 60 cm; JL Behv-LAG-8, Shanghai Jiliang Software Technology Company Limited Shanghai, China) equipped with photocell beams for a period of 1 h. Locomotor activity was detected through beam-interruptions, and distance traveled and time in the central area were recorded. Rats with the highest third and lowest third of total distance traveled in the test were classified as HR group and LR group, respectively (Kabbaj et al., 2000; Jama et al., 2008).

### Western Blot Assay

The protocols for sample preparation and Western blot were based on previous studies (Xu et al., 2017; Han et al., 2019). One day after the behavioral tests, the rats were decapitated and the brains were rapidly extracted and frozen in −60°C *N*-hexane. The brains were then transferred to a −80°C freezer. Bilateral tissue punches (12-gauge) were collected from the PrL and homogenized with RIPA lysis buffer (Applygen Technology, Beijing, China) with enzyme inhibitor cocktail (Applygen Technology, Beijing, China). The cell lysates were centrifuged at 12,000× *g* for 15 min. The protein concentrations in all of the samples were determined using the BCA assay kit (Applygen Technology, Beijing, China). The samples were diluted with RIPA lysis buffer to equalize protein concentrations. Equal amounts of samples (30 μg) were subjected to 8% sodium dodecyl sulfate-polyacrylamide gel electrophoresis. The primary antibodies were the following: mouse anti-neurocan (1:2,000, Sigma-Aldrich, St. Louis, MO, USA, catalog no. N-0913), rabbit anti-aggrecan (1:2,000, Sigma-Aldrich, St. Louis, MO, USA, catalog no. SAB4500662), mouse anti-tenascin-R (1:2,000, SySy Goettingen, Germany, catalog no. 217011), and mouse anti-β-actin (1:2,000, Sigma-Aldrich, St. Louis, MO, USA, catalog no. A5316). Horseradish peroxidase-conjugated secondary antibody was used (1:2,000, goat anti-rabbit and goat anti-mouse IgG, ZSGB-BIO, China). The levels of all of the molecules were normalized to the level of β-actin. The western blot experiments were carried out twice in duplicates. Band intensities were quantified by two observers who were blind to the experimental groups using NIH ImageJ software.

### Immunohistochemistry

One day after the behavioral tests, the rats were perfused with 4% paraformaldehyde, and brains were removed and post-fixed in 4% paraformaldehyde for 24 h. The brains were then dehydrated in 30% sucrose (w/v) in 0.1 M phosphate buffer and stored at −80°C until analysis. Briefly, the brains were coronally sectioned with a microtome into 25 μm thick sections, and five to six sections apart spanning the rostrocaudal axis of the PrL (within 4.2 mm to 2.52 mm from Bregma) from each rat were collected and stained. All of the sections were washed in phosphate-buffered saline (0.1 M PBS) 5 min each time for three times and then soaked in blocking solution [1% bovine serum albumin (BSA, Amresco, WA, catalog no.0175), 3% donkey serum (Applygen Technology, Beijing, China), and 0.3% (v/v) Triton X-100 (Sigma-Aldrich, St. Louis, MO, USA) in PBS, pH 7.4] for 1 h at 25°C. The sections were incubated overnight at 4°C in biotin-conjugated lectin *Wisteria floribunda* agglutinin (WFA, 10 mg/mL, Sigma-Aldrich, St. Louis, MO, USA, catalog no. L1516) and primary rabbit anti-NeuN antibody (1:500, Abcam, Cambridge, UK, catalog no. ab177487). All of the sections were then washed three times in PBS and then incubated in FITC-conjugated streptavidin (10 mg/mL, Sigma-Aldrich, St. Louis, MO, USA, catalog no. S3762) and donkey anti-rabbit IgG H&L (405; 1:500, Abcam, Cambridge, UK, catalog no. ab175651).

For quantification, a fluorescence microscope (Olympus VS120) with an image-analysis program (NIH ImageJ software) was used to measure the number of WFA^+^ PNNs, NeuN^+^ cells, and their colocalization. Cells were counted in two randomly selected fields within a 6.6-fold defined area in the PrL in the control and experimental groups using NIH ImageJ software. The size of sampled areas for cell quantifications of each brain region from each section was 0.687 × 0.502 mm^2^, for a total area of 0.345 mm^2^. The borders of the PrL were defined by reference to the rat brain atlas (Paxinos and Watson, 2007) using the NeuN immunostaining. Five to six sections for each rat were selected, and the average cell numbers on either side of the target brain region from all sections was taken as the number of immunoreactive cells for each rat. Scoring for all determinations was performed by an investigator who was blind to the groups.

### Statistical Analysis

The statistical analysis was performed using GraphPad Prism 7 software. The data are expressed as mean ± SEM and were analyzed using one- or two-way analysis of variance (ANOVA) with appropriate between- and within-group factors for the different experiments, followed by Tukey’s *post hoc* tests. If two groups displayed normal distributions and had equal variance, an unpaired *t*-test was used; otherwise non-parametric Mann–Whitney *U* test was used. Values of *p* < 0.05 were considered statistically significant.

## Results

### Depressive-Like Behavior Induced by 30 Days of CUMS Was Coincident With Decreased Density of PNNs

**Figure 1:**
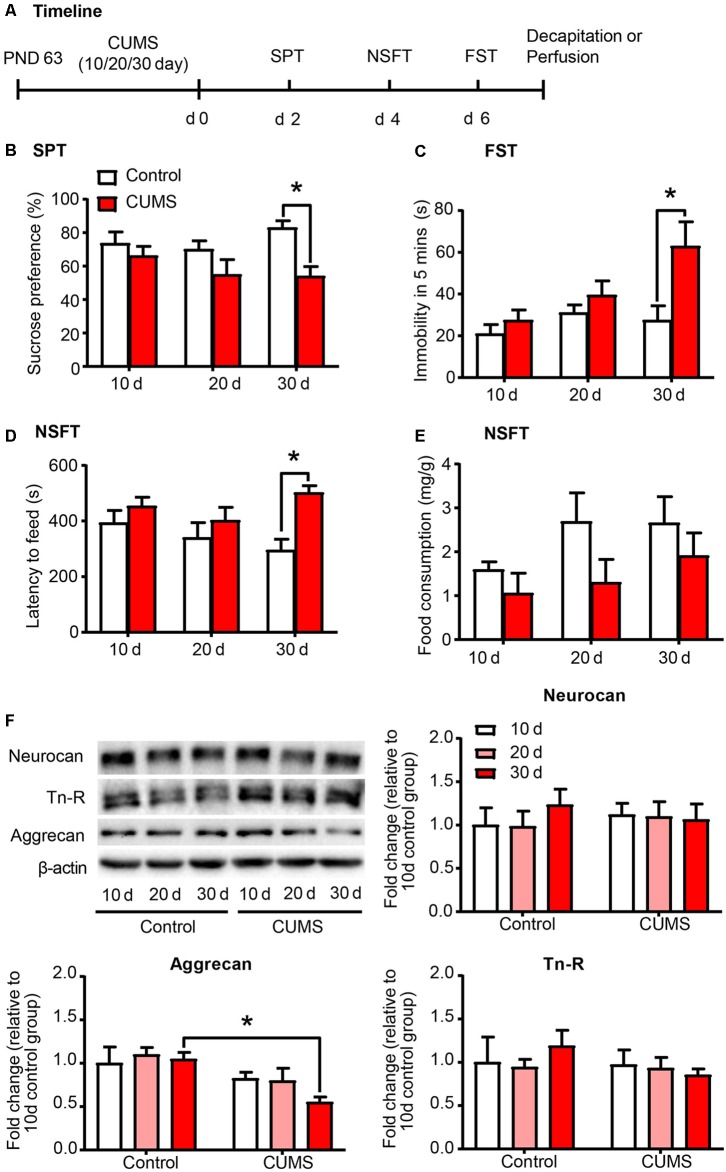
Depressive-like behavior induced by 30 days of chronic unpredictable mild stress (CUMS) was coincident with decreased aggrecan expression in the prelimbic cortex (PrL). **(A)** Timeline of the experimental procedure (*n* = 8–10 per group). **(B)** Sucrose preference in the sucrose preference test (SPT). **(C)** Immobility time in the forced swim test (FST). **(D)** Latency to feed and **(E)** food intake in home cage in the novelty-suppressed feeding test (NSFT) after 10 days, 20 days or 30 days of chronic unpredictable mile stress (CUMS). **(F)** Representative Western blots and quantification of fold changes in protein levels of PNNs components including neurocan, aggrecan and tenascin-R (Tn-R) in the PrL (*n* = 7 per group). The data are expressed as mean ± SEM. *p < 0.05. PND, postnatal day.

Exposure to environmental stressors may promote the development of depression. Here we sought to identify the development of depressive-like behaviors by subjecting rats to 10 days, 20 days and 30 days of CUMS, which is the most commonly used and acknowledged rodent model of depression (Antoniuk et al., 2019). After CUMS, the rats underwent several depressive-like and anxiety-like behavioral tests subsequently, including SPT, NSFT and FST (*n* = 8–10 per group; Figure 1A). Then the expression of PNN components, including aggrecan, neurocan and Tn-R in PrL were assessed by Western blot (*n* = 7 per group). The results revealed that 30 days, but not 10 days or 20 days, of CUMS induced depressive- and anxiety-like behaviors in rats, as indicated by the decrease in sucrose preference in the SPT (two-way ANOVA, stress × time interaction, *F*_(2,49)_ = 1.368, *p* > 0.05; main effect of stress, *F*_(1,49)_ = 10.690, *p* < 0.01; main effect of time, *F*_(2,49)_ = 0.796, *p* > 0.05; *post hoc* test, 30 days Control vs. 30 days CUMS, *p* < 0.05; Figure 1B), the increase in immobility time in the FST (two-way ANOVA, stress × time interaction, *F*_(2,49)_ = 2.800, *p* = 0.071, main effect of stress, *F*_(1,49)_ = 9.723, *p* < 0.01; main effect of time, *F*_(2,49)_ = 4.862, *p* < 0.05; *post hoc* test, 30 days Control vs. 30 days CUMS, *p* < 0.05; Figure 1C) and the increase in latency to feeding in the NSFT (two-way ANOVA, stress × time interaction, *F*_(2,49)_ = 1.796, *p* > 0.05, main effect of stress, *F*_(1,49)_ = 9.629, *p* < 0.01; main effect of time, *F*_(2,49)_ = 0.787, *p >* 0.05; *post hoc* test, 30 days Control vs. 30 days CUMS, *p* < 0.05; Figure 1D) without affecting total food consumption (Figure 1E). The expression of aggrecan (two-way ANOVA, stress × time interaction, *F*_(2,36)_ = 0.9925, *p* > 0.05, main effect of stress, *F*_(1,36)_ = 11.99, *p* < 0.01; main effect of time, *F*_(2,49)_ = 0.9308, *p* > 0.05; *post hoc* test, 30 days Control vs. 30 days CUMS, *p* < 0.05; Figure 1F) is decreased after 30 days of CUMS, but chronic stress had no significant effects on the protein levels of neurocan and Tn-R in the PrL. These results suggest that PNNs in the PrL may be involved in the depressive-like behaviors induced by chronic stress.

### CUMS-Vulnerable Rats Exerted Lower Density of PNNs in the PrL Than Resilient Rats

**Figure 2:**
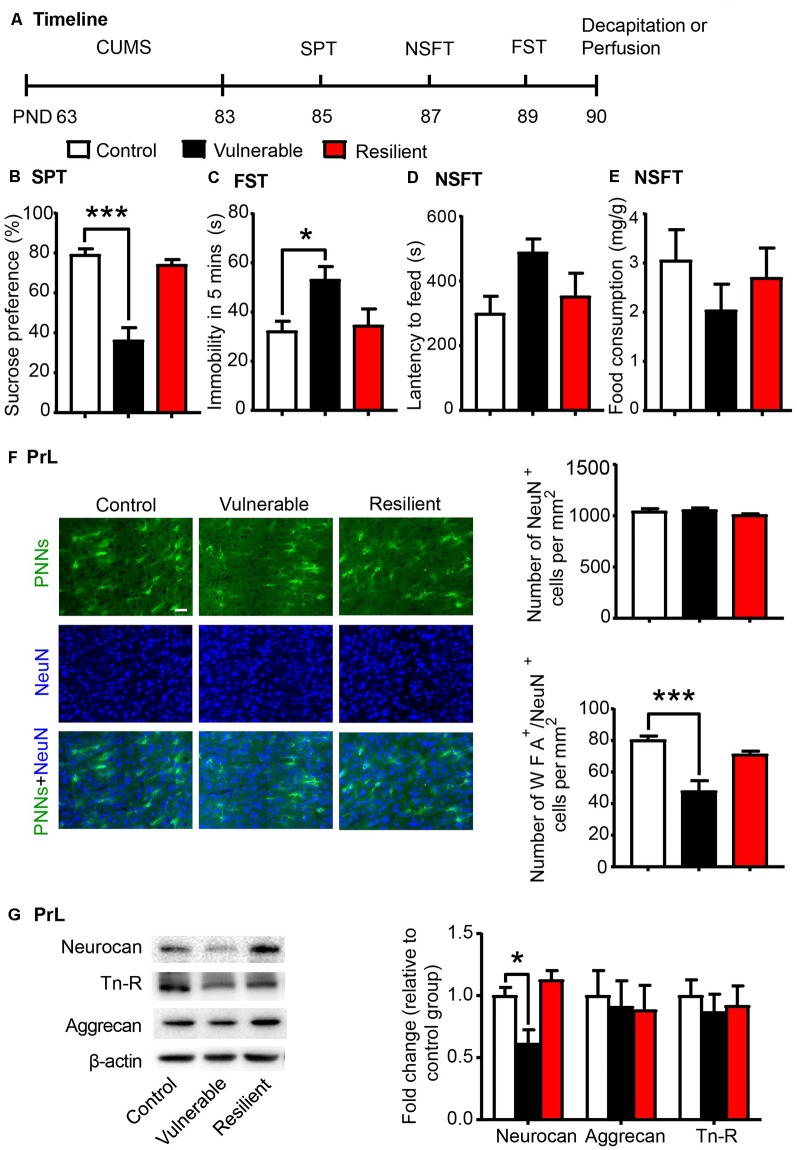
CUMS-vulnerable rats exhibited a lower density of Perineuronal nets (PNNs) in the PrL compared to control and resilient rats. **(A)** Timeline of the experimental procedure (*n* = 8 per group). **(B)** Sucrose preference in the SPT. **(C)** Immobility time in the FST. **(D)** Latency to feed and **(E)** food intake in home cage in the NSFT after 20 days of chronic unpredictable mile stress (CUMS). The vulnerable rats displayed anhedonia and helpless behavior, while resilient rats did not. **(F)** Representative images of immunofluorescence staining of PNNs and quantification of NeuN^+^ and WFA^+^/NeuN^+^ cells in the PrL (*n* = 6 per group). Scale bar = 50 μm. **(G)** Representative western blots and quantification of fold changes in protein levels of PNNs components including neurocan, aggrecan and tenascin-R (Tn-R) in the PrL (*n* = 8 per group). The data are expressed as mean ± SEM. *p < 0.05, ***p < 0.001. PND, postnatal day.

Individual differences in responses to chronic stress have been reported, and stress exerts differential behavioral and neurochemical effects on CUMS-vulnerable and resilient animals (Bisgaard et al., 2007; Bergström et al., 2008). Similarly, we separated rats into vulnerable and resilient sub-populations based on the sucrose preference in the SPT (*n* = 8 per group; Figure 2A). After 20 days of CUMS, rats that exhibited sucrose preference in the highest 30% of the sample were classified as resilient group, and rats that exhibited sucrose preference in the lowest 30% of the sample were classified as vulnerable group (Bergström et al., 2008; Taliaz et al., 2011; Li et al., 2014). The vulnerable rats, but not the resilient rats, exhibited depressive- and anxiety-like behaviors after 20 days of CUMS, as indicated by the decrease in sucrose preference in the SPT (one-way ANOVA, *F*_(2,21)_ = 30.61, *p* < 0.001; *post hoc* test, Control vs. Vulnerable, *p* < 0.001; Figure 2B), the increase in immobility time in the FST (one-way ANOVA, *F*_(2,21)_ = 4.612, *p* < 0.05; *post hoc* test, Control vs. Vulnerable, *p* < 0.05; Figure 2C) and the increase in latency to feeding in the NSFT (one-way ANOVA, *F*_(2,21)_ = 3.007, *p* = 0.071; Figure 2D) without affecting total food consumption (Figure 2E).

The lectin WFA binds specifically to the *N*-acetyl-D-galactosamine on terminal ends of chondroitin sulfate chains and is commonly used to detect PNNs in the brain using immunohistochemistry (Reichelt et al., 2019; Testa et al., 2019). Then we investigated the density of WFA-labeled PNNs (*n* = 6 per group) and the expression of PNN components (*n* = 8 per group) in the PrL after the behavioral tests were conducted. The localization of the selected PrL area used for PNN quantification was indicated in Supplementary Figure S1. The vulnerable group displayed decreased number of WFA-labeled PNNs (one-way ANOVA, *F*_(2,15)_ = 14.26, *p* < 0.001; Supplementary Figure S2A) and its colocalization with neuronal marker NeuN (one-way ANOVA, *F*_(2,15)_ = 15.92, *p* < 0.001; *post hoc* test, Control vs. Vulnerable, *p* < 0.001; Figure 2F) in the PrL compared to the control and resilient groups. There were no significant differences in number of NeuN-positive cells among three groups (one-way ANOVA, *F*_(2,15)_ = 1.718, *p* > 0.05; Figure 2F). The expression of neurocan (one-way ANOVA, *F*_(2,21)_ = 9.589, *p* < 0.01; *post hoc* test, Control vs. Vulnerable, *p* < 0.05; Figure 2G) was also decreased in the PrL of vulnerable rats, but there was no significant difference in the protein levels of aggrecan and Tn-R among the three groups.

These results indicate that decreased density of PNNs in the PrL may be related to the susceptibility to chronic stress and promote the development of depressive-like behaviors in young-aged rats.

### PNN Density and Component Expression Levels Were Associated With Individual Differences in Novelty Seeking Behavior

**Figure 3:**
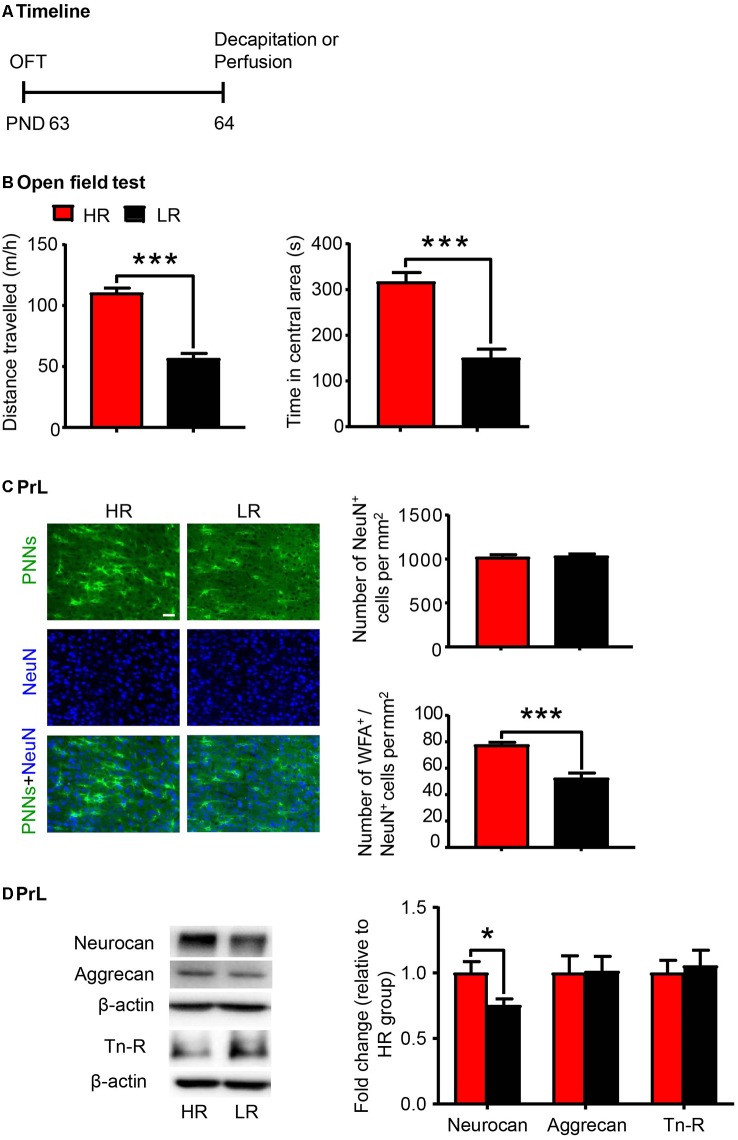
PNN density and component expression levels were associated with individual differences in locomotor response to novelty. **(A)** Timeline of the experimental procedure (*n* = 20 per group). **(B)** Total distance traveled and time spent in a central area in the open-field test (OFT). **(C)** Representative images of immunofluorescence staining of PNNs and quantification of NeuN^+^ and WFA^+^/NeuN^+^ cells in the PrL (*n* = 6 per group). Scale bar = 50 μm. **(D)** Representative western blots and quantification of fold changes in protein levels of PNNs components including neurocan, aggrecan and tenascin-R (Tn-R) in the PrL (*n* = 8 per group). The data are expressed as mean ± SEM. *p < 0.05, ***p < 0.001. HR, high responding phenotype; LR, low responding phenotype. PND, postnatal day.

Individual differences in locomotor response to novelty is an important factor to predict vulnerability to stress-induced depression (Kabbaj et al., 2000; Weger and Sandi, 2018) and drug addiction (Piazza et al., 1989; Kalinichev et al., 2004). Here a separate group of rats were subjected to the OFT and divided into HR and LR phenotypes according to the distance traveled (*n* = 20 per group; Figure 3A). The HR rats exhibited significantly higher locomotor activity (*t*-test, *t*_(38)_ = 9.189, *p* < 0.001; Figure 3B) and more time spent in the central area (Mann–Whitney *U*, *p* < 0.001; Figure 3B) in the OFT compared to the LR rats. We next evaluated the density of PNNs (*n* = 6 per group) and the expression of PNN components (*n* = 8 per group) in the PrL. We found that the number of WFA-positive cells (*t*-test, *t*_(10)_ = 3.767, *p* < 0.01; Supplementary Figure S2B) and its colocalization with NeuN (*t*-test, *t*_(10)_ = 5.514, *p* < 0.001; Figure 3C) were decreased in the PrL of LR rats. The number of NeuN-positive cells was not significantly different between HR and LR groups (*t*-test, *t*_(10)_ = 0.329, *p* > 0.05; Figure 3C). The protein level of neurocan (*t*-test, *t*_(14)_ = 2.509, *p* < 0.05; Figure 3D) was also decreased in the LR group compared to HR group, but the expression levels of aggrecan and Tn-R showed no significant difference between groups. These results suggest that the PNN density in the PrL can be considered as an index of individual differences in novelty-seeking behavior.

### Low-Responding Phenotype With Low Density of PNNs Predicted the Vulnerability to CUMS

**Figure 4:**
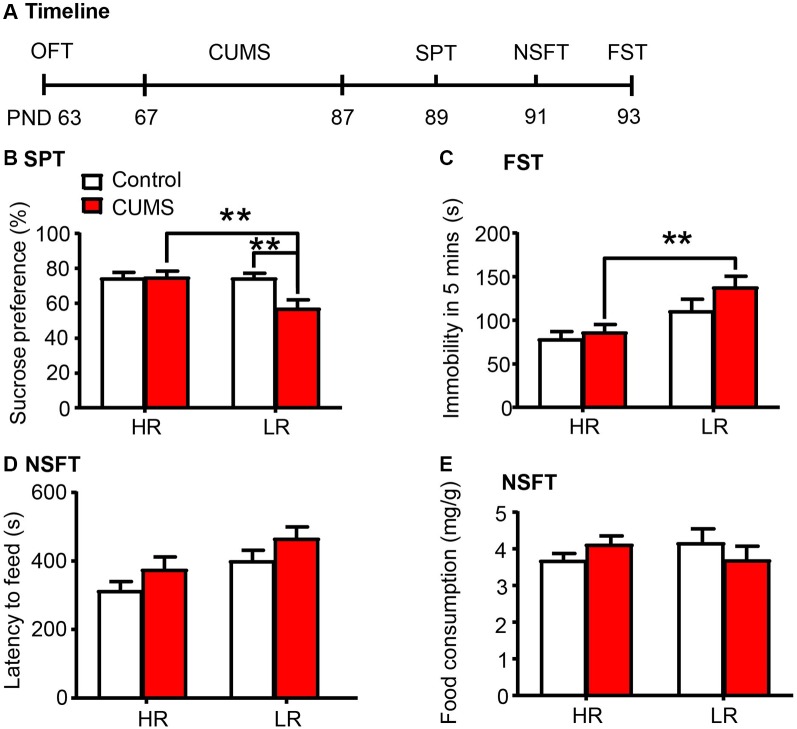
Low responding phenotype with a low density of PNNs predicted the vulnerability to CUMS. **(A)** Timeline of the experimental procedure (*n* = 15 per group). **(B)** Sucrose preference in the SPT. **(C)** Immobility time in the FST. **(D)** Latency to feed and **(E)** food intake in the home cage in the NSFT. The LR rats exhibited depressive-like behaviors after 20 days of chronic unpredictable mile stress (CUMS), while the HR rats did not. The data are expressed as mean ± SEM. **p < 0.01. HR, high responding phenotype; LR, low responding phenotype. PND, postnatal day.

Here we investigated whether individual differences in locomotor response to novelty are related to depressive- and anxiety-like behaviors in rats (*n* = 15 per group; Figure 4A). We found that the LR rats exhibited increased vulnerability to 20 days of CUMS, as indicated by the decrease in sucrose preference in the SPT (two-way ANOVA, phenotype × stress interaction, *F*_(1,56)_ = 5.868, *p* < 0.05, main effect of phenotype, *F*_(1,56)_ = 5.805, *p* < 0.05; main effect of stress, *F*_(1,56)_ = 5.175, *p* < 0.05; *post hoc* test, HR Stress vs. LR Stress, *p* < 0.01, LR Control vs. LR Stress *p* < 0.01; Figure 4B), the increase in immobility time in the FST (two-way ANOVA, phenotype × stress interaction, *F*_(1,56)_ = 0.789, *p* > 0.05, main effect of phenotype, *F*_(1,56)_ = 14.38, *p* < 0.001; main effect of stress, *F*_(1,56)_ = 2.538, *p* > 0.05; *post hoc* test, HR Stress vs. LR Stress, *p* < 0.01; Figure 4C) and the increase in latency to feeding in the NSFT (two-way ANOVA, phenotype × stress interaction, *F*_(1,56)_ = 0.004, *p* > 0.05, main effect of phenotype, *F*_(1,56)_ = 7.838, *p* < 0.01; main effect of stress, *F*_(1,56)_ = 4.028, *p* < 0.05; Figure 4D) without affecting total food consumption (Figure 4E), compared to HR rats. Altogether, these results indicate that the predisposition to depression in LR rats may be attributed to the low PNN density and neurocan expression in the PrL.

## Discussion

CUMS represents a significant contributor to the development of depressive-like and anxiety-like behaviors. In the present study, we found that depressive-like behaviors were induced by 30 days, but not 10 days or 20 days, of CUMS. These behavioral effects were accompanied by decreases in the protein level of aggrecan, which is a core component of the condensed glycosaminoglycan-rich ECM structures termed PNNs. Aggrecan has been shown to regulate neuronal plasticity, and its expression is specifically decreased after sensory deprivation through whisker trimming (McRae et al., 2007; Rowlands et al., 2018). Human postmortem studies revealed marked abnormalities of aggrecan and PV-expressing neurons in schizophrenia and bipolar disorder (Pantazopoulos et al., 2015; Steullet et al., 2018). Preclinical studies also showed that early life stress or chronic stress during adulthood altered PNN numbers in multiple brain regions including prefrontal cortex (Castillo-Gómez et al., 2017; de Araújo Costa Folha et al., 2017; Pesarico et al., 2019). The PNN assembly and its molecular components may play a critical role in stress response.

PNNs are complex ECM structures with heterogeneous composition and have been shown to be involved in synaptic plasticity, neuronal communication and neuroprotective function (Reichelt et al., 2019; Testa et al., 2019). PNNs are mainly constituted by hyaluronic acid, Tn-R and the lectican family of CSPGs (i.e., aggrecan, neurocan, brevican, and versican). TnR and aggrecan are the structural proteins of PNNs, and TnR promotes the formation of PNNs by clustering of aggrecan (Morawski et al., 2014). TnR-deficient mice displayed abnormal postnatal development of PNNs and exhibited increased anxiety-like behavior and reduced exploration in the open field and elevated plus-maze tests (Brückner et al., 2000; Freitag et al., 2003). Genetic, pharmacological or environmental disruption of PNN assembly contributes to the pathogenesis of epilepsy, schizophrenia and other psychiatric disorders (Bitanihirwe et al., 2016; Testa et al., 2019).

Growing evidence supports individual variations in susceptibility to stress, and understanding the genetic, environmental, and developmental mechanisms that underlie stress resilience is of great significance to the prevention and treatment of depression (Feder et al., 2009; Southwick and Charney, 2012). In the present study, we utilized the CUMS model to investigate the biological basis of individual differences in response to stress. We revealed variances in several behavioral outcomes after 20 days of CUMS. The vulnerable rats exhibited anhedonia, hopelessness and anxiety-like behaviors after chronic stress, while the resilient rats did not. Furthermore, we found that PNN density and neurocan expression were decreased in the PrL of vulnerable rats but not in control and resilient rats.

Previous studies identify high anxiety trait as a vulnerable phenotype for stress-induced depression (Weger and Sandi, 2018). Individual variations in novelty-seeking behavior, such as differences in locomotor activity during exposure to a novel environment, have also been reported to predict the predisposition to stress-related mood disorders and drug-related behavior (Piazza et al., 1989; Kabbaj et al., 2000; Duclot et al., 2011; Carreira et al., 2017). Therefore, in the present study, we divided rats into HR and LR sub-populations according to the total distance traveled in the OFT. We found that the number of WFA-positive PNNs and the expression level of neurocan was lower in the PrL of LR rats compared to HR rats. Moreover, the LR rats were more susceptible to depressive-like behaviors induced by 20 days of CUMS compared to HR rats, indicating that low PNN density in the PrL of LR rats may promote the development of depressive-like behaviors in rats. Inconsistent with our findings, previous studies showed that HR rats are vulnerable to social defeat, while LR rats are not (Duclot et al., 2011; Duclot and Kabbaj, 2013). The effect of HR and LR phenotype on stress vulnerability may be affected by different types of stress exposure. During the social defeat stress paradigm, the HR rats with high spontaneous activity can be attacked more often by attackers, which may cause higher vulnerability to social defeat, compared to LR rats with low spontaneous activity (Duclot et al., 2011; Duclot and Kabbaj, 2013). Additionally, although CUMS and novelty phenotype affect the protein levels of aggrecan or neurocan in the PrL, it is still unknown whether they affect PNN structure and function in a different way.

Genetic studies in humans revealed that the variation of NCAN, a gene encoding PNN component protein neurocan, is a common risk factor for schizophrenia and bipolar disorder (Mühleisen et al., 2012; Oruč et al., 2012; Schultz et al., 2014). Neurocan has also been shown to regulate brain development, dendritic spine remodeling and the function of GABAergic interneurons (Zhou et al., 2001; Mohan et al., 2018; Sullivan et al., 2018). The assembly and function of PNNs are disrupted when these component proteins are hydrolyzed or the structures are destroyed, which could ultimately lead to negative consequences and pathophysiology of neuropsychiatric disorders (Wen et al., 2018; Testa et al., 2019). Altogether, our findings indicate that the reduction in neurocan expression in CUMS-vulnerable or LR rats may contribute to decreased density of PNNs in the PrL and promote the occurrence of depressive-like behaviors in young-aged rats. The low density of PNN may serve as an innate biological trait or potential endophenotype that can predict susceptibility to stress. However, the present study could not conclude the causal relationship and correlation between the alteration in PNN density and behaviors. Further studies are needed to elucidate the mechanism underlying the effects of altered PNN density on the specific neuronal function and behavioral abnormalities.

PNNs preferentially envelop PV-expressing neurons in many cortical regions, which could protect interneurons from environmental and oxidative stress damage during critical periods of brain plasticity (Cabungcal et al., 2013; Reichelt et al., 2019). Disruption of the PNNs or enzymatic removal of the ECM reduced glutamatergic input to PV-expressing interneurons, reduced PV excitability, increased gamma activity and regulated GABAergic transmission (Frischknecht et al., 2009; Lensjö et al., 2017; Hayani et al., 2018; Hirono et al., 2018). The expression levels of PNN components and PV neurons are reduced, and the integrity of PNNs is impaired in many psychiatric disorders such as schizophrenia and mood disorders (Pantazopoulos and Berretta, 2016; Testa et al., 2019). Additionally, the perisynaptic or perinodal ECM also plays an important role in the regulation of synaptic plasticity and neuronal function and may be involved in the pathophysiology of brain disorders (Faissner et al., 2010; Fawcett et al., 2019). The present study provides evidence supporting the association between PNN density in the PrL and depression. The protection of PNNs or perisynaptic ECM for the PV interneurons may be impaired under stress conditions, thus contributing to the alterations in prefrontal cortex function and the development of depressive-like behaviors.

In summary, the present study focuses on investigating the association between PNN assembly and stress-induced depression. The results demonstrate that the low density and component protein expression of PNNs in the PrL of vulnerable and LR rats may predict individual susceptibility to chronic stress and promote the development of depressive-like behaviors. Further studies are needed to elucidate the contributions of PNNs in the pathogenesis of depression *via* manipulating specific PNN components or disrupting its structure.

## Data Availability Statement

The raw data supporting the conclusions of this article will be made available by the authors, without undue reservation, to any qualified researcher.

## Ethics Statement

The animal study was reviewed and approved by the Biomedical Ethics Committee for Animal Use and Protection of Peking University and carried out in accordance with the National Institutes of Health Guide for the Care and Use of Laboratory Animals.

## Author Contributions

YH and JS designed the study. ZY, NC, DH, WC, and YY performed the experiments. ZY, DH, WZ, SM, and YH analyzed and interpreted the data. ZY, NC, SM, LL, YH, and JS wrote and revised the article.

## Conflict of Interest

The authors declare that the research was conducted in the absence of any commercial or financial relationships that could be construed as a potential conflict of interest.
