# Supplementary figures and images for: Decreased Density of Perineuronal Net in Prelimbic Cortex Is Linked to Depressive-Like Behavior in Young-Aged Rats
Source: Front Mol Neurosci. 2020 Jan 28;13:4. doi: 10.3389/fnmol.2020.00004 (PMC7025547; doi:10.3389/fnmol.2020.00004)

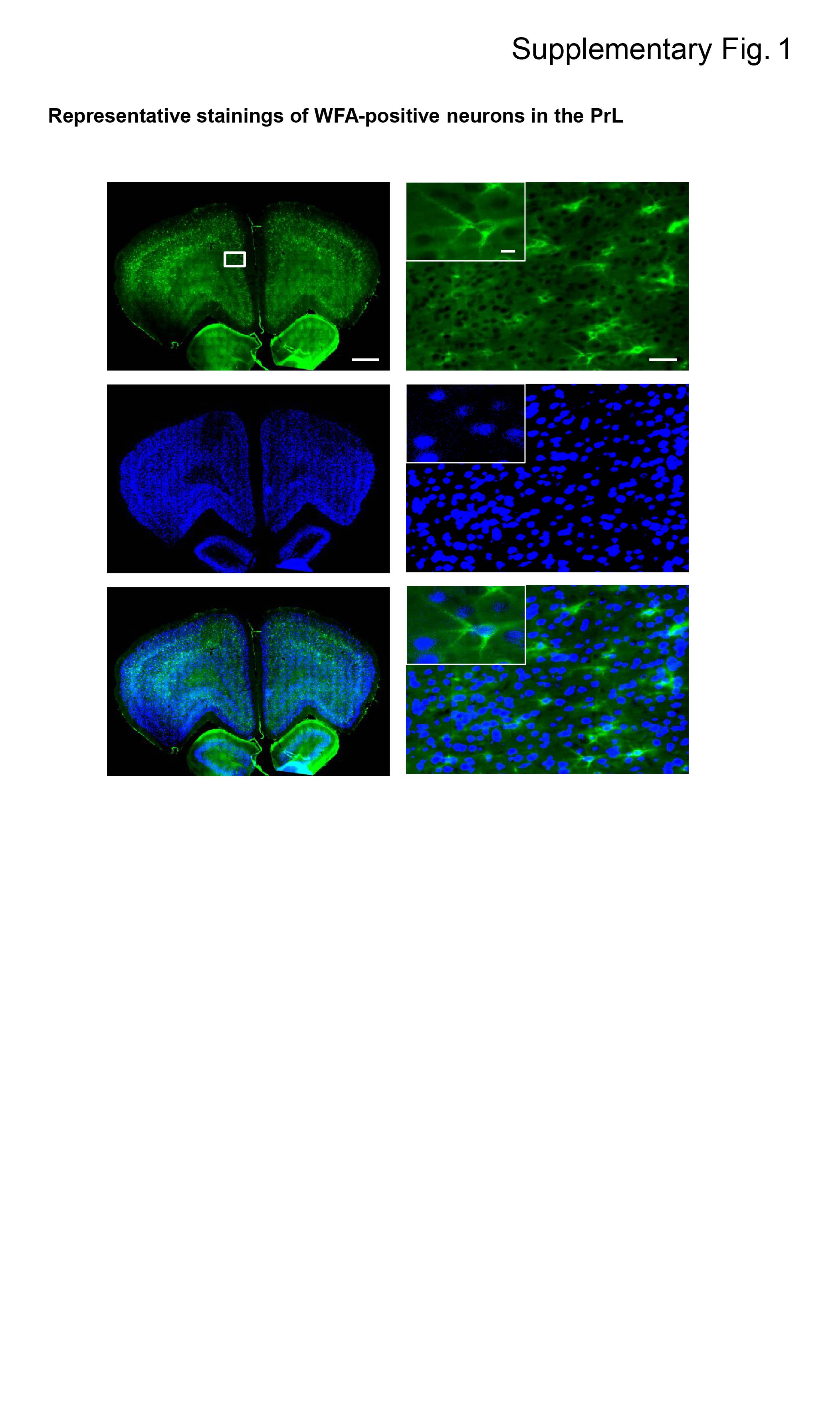

Supplement: Supplementary file 1 [file Image_1.JPEG]

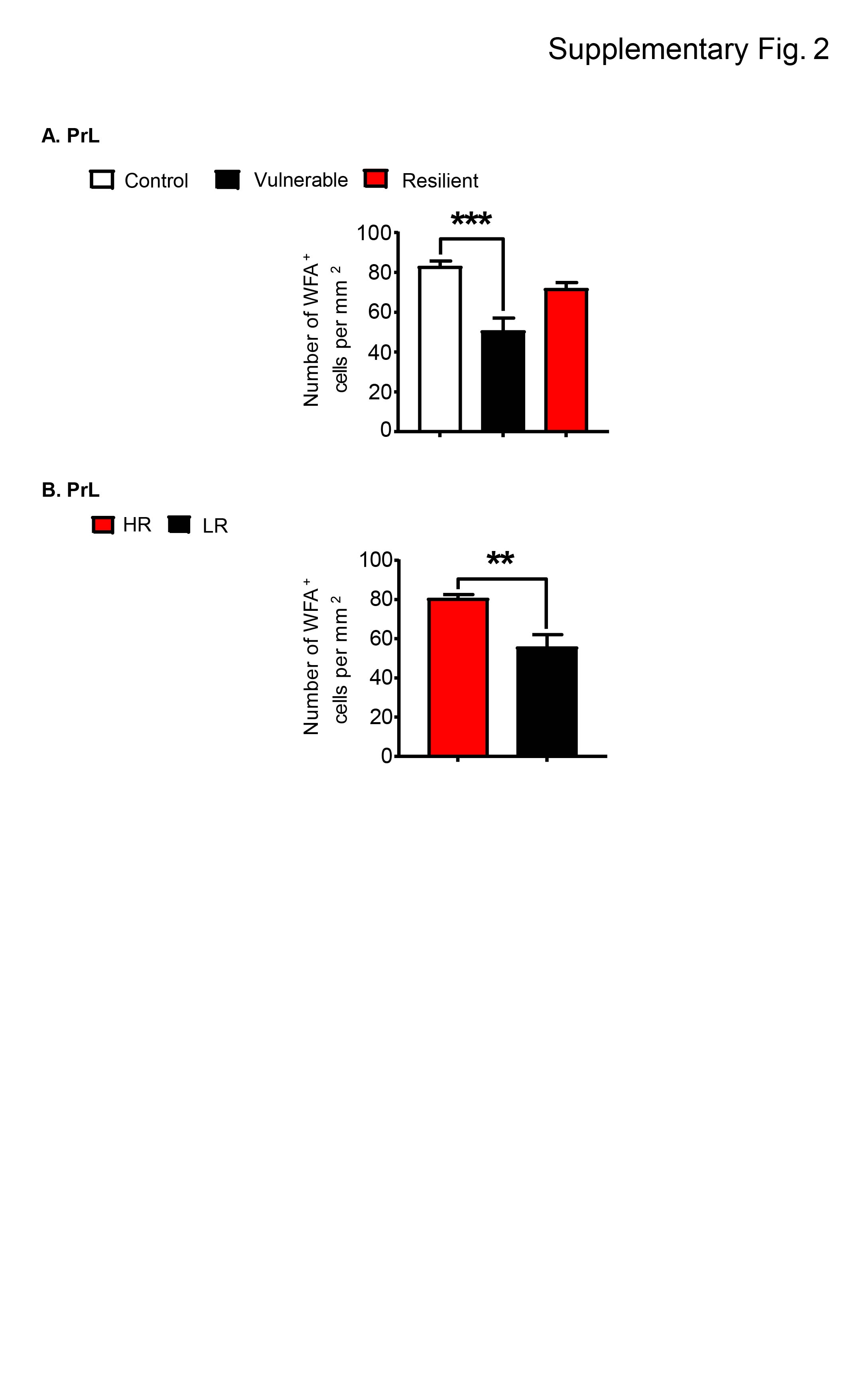

Supplement: Supplementary file 2 [file Image_2.JPEG]
