# Supplementary material for: Decreased Density of Perineuronal Net in Prelimbic Cortex Is Linked to Depressive-Like Behavior in Young-Aged Rats
Source: Front Mol Neurosci. 2020 Jan 28;13:4. doi: 10.3389/fnmol.2020.00004 (PMC7025547; doi:10.3389/fnmol.2020.00004)
Supplement: Supplementary file 3 [file Table_1.doc]

Supplementary Information

Supplementary Figure 1. Representative images of immunofluorescence staining of perineuronal nets in rat brain and the localization of selected prelimbic cortex (PrL) area. Left panels, Scale bar = 1 cm; Right panels, Scale bar = 50 μm. Enlarged images of representative PNNs (Scale bar = 10 μm) are inserted in the Right panels.

Supplementary Figure 2. Quantification of WFA-positive cells in the PrL. (A) CUMS-vulnerable rats exhibited lower density of PNNs in the PrL compared to control and resilient rats (*n* = 6 per group). (B) Low responding (LR) phenotype rats exhibited lower density of PNNs in the PrL compared to high responding (HR) phenotype (*n* = 6 per group). ***p* < 0.01, ****p* < 0.001.
